# Applying General Strain Theory to the Relationship Between Strain from Another Person’s Gambling Behavior and Gambling Disorder

**DOI:** 10.1007/s10899-024-10351-1

**Published:** 2024-08-14

**Authors:** Michelle L. Malkin

**Affiliations:** Department of Criminal Justice and Criminology, Gambling Research & Policy Initiative, East Carolina University, 238 Rivers Building, Greenville, NC 27858 USA

**Keywords:** Gambling disorder, General strain theory, Gender, Problem gambling, Strain, Affected others, Spouse

## Abstract

Prior research has identified a host of factors that increase the likelihood that an individual will develop problem gambling, clinically diagnosed as Gambling Disorder (GD), most of which would be identified by criminologists as “strains” under the framework of General Strain Theory (GST). This study utilizes propositions from GST to determine whether strain from another person’s gambling may be related to why people develop GD and whether gender is a moderating factor in this relationship. Secondary data is analyzed to assess levels of strain individuals experience from another person’s gambling behavior, its relationship to the individual’s risk of Gambling Disorder, and the role gender plays in this relationship. Findings demonstrate a relationship between the strain from the perceived problem gambling of someone with a close relationship and having a gambling disorder. Experiencing strain from a spouse/partner who is perceived as a problem gambler has the strongest correlation with an individual also having Gambling Disorder. Considering gender as a moderating factor, this effect was stronger on men than women, calling into question the strong belief that it is primarily women who gamble to escape problems.

## Introduction

Broad legalization of gambling throughout the country resulted in increased negative consequences related to gambling (American Psychiatric Association, 2013; Clark & Walker, 2009; Crofts, 2003). One such consequence is a possible growing addiction to gambling, referred to medically as *gambling disorder* but more commonly called “problem gambling” (American Psychiatric Association, 2013; Welte et al., 2014). Problem gambling results in persistent and recurrent maladaptive behavior that may disrupt all aspects of an individual’s life, including personal, family, and/or vocational pursuits. With at least 2% of the American adult population experiencing problem gambling and the rapid expansion of gambling opportunities throughout the United States, problem gambling has negative implications for those suffering from the disorder, as well as society as a whole (Petry et al., 2014; Shaffer et al., 1999).

Gender is an important factor in understanding the pathways, effects, and prevalence of gambling disorder (Afifi et al., 2010). Specifically, women may become disordered gamblers for different reasons than men (Blanco et al., 2006; Sacco et al., 2011; Weatherly & Cookman, 2014). For example, women have been characterized as primarily “escape” gamblers, gambling to hide from problems; whereas men have been described as “action” gamblers who gamble to boost adrenaline (Blanco et al., 2006; Sacco et al., 2011; Weatherly & Cookman, 2014). Based on these characterizations, the impact of strain on problem gambling may differ for women and men in that strain may represent a larger reason women gamble and potentially become disordered gamblers than men.

Although gambling disorder is strongly related to criminal behavior, little criminological theory has been applied to this issue. One theory that may provide a good fit is General Strain Theory (GST), given its prior use to explain other forms of addiction (Baron & Hartnagel, 1997; Ford & Schroeder 2009; Piquero et al., 2010). GST posits that addiction may be due to negative emotions caused by certain types of strain (Agnew, 1992).

This study applies GST as a potential explanation for problem gambling. It examines the role of gender as a moderating factor through secondary analysis of data collected from the Social and Economic Impacts of Gambling in Massachusetts (SEIGMA) study (SEIGMA, 2020). These data provide a unique opportunity to examine strain and its relationship to gambling disorder, as few gambling studies have been this comprehensive. Of the 14,569 total participants, 5,852 were identified as participating in some form of gambling at least monthly within the preceding year, and 446 were scored on the assessment as having a gambling problem (Williams et al., 2012).

This study furthers the existing understanding of the relationship between GST, gambling disorder, and the role gender plays in this relationship. Additional research is needed, as only five prior studies specifically considered this relationship (Cheung, 2015; Eitle & Taylor, 2010; Greco & Curci, 2016; Kabiri et al., 2020; Man & Cheung, 2022). This is only the second study to examine this issue with a population from the United States and one of the only studies to consider gender as a moderator. Specifically relevant, Cheung (2015) studied whether strain from couple dynamics could predict gambling problems in China. The study found that strain experienced by a male partner could predict both his and his wife’s likelihood of developing into problem gambling. In contrast, strain by the female partner could predict her likelihood of developing a gambling problem, however, there was no association with her husband’s gambling behavior. Using the same data, Man and Cheung (2022) assessed gambling disorder across gender and gender norms. They found that gender norms increased the effect of strain on men but could mitigate the strain in women, resulting in gender norm strain being more likely to result in gambling disorder in men. Therefore, the current study attempts to answer whether an individual’s strain from the perceived belief that a friend or family member gambles too much will be related to gambling disorder. Based on Cheung’s (2015) and Man and Cheung’s (2022) studies out of China, it was anticipated that strain from a friend or family member’s problem gambling would increase the prevalence of gambling disorder. Based on the specific findings from Cheung’s (2015) study and the historical belief that women may gamble to escape, it was anticipated that the effect would be stronger on women.

### Gambling Disorder, Gender, and General Strain Theory

The following provides an overview of prior research on gambling disorder and General Strain Theory (GST). It starts by exploring concepts related to Gambling Disorder. Next, relevant literature related to problem gambling and gender is discussed. Finally, research related to GST is introduced.

### Understanding Gambling Disorder

Relaxed gambling laws, which started in the late 20th Century, resulted in and a massive spread of many types of legal gambling throughout the country (Clark & Walker, 2009; National Opinion Research Center, 1999; Walker, 2007; Welte et al., 2014; Williams et al., 2012). Consequently, this has led to an increase in people who are addicted to gambling.^1^ Problem gambling can be placed on a continuum that ranges from probable and moderate addiction to severe addiction (Greco & Curci, 2016; Toce-Gerstein et al., 2003). Approximately 25 years ago, the National Gambling Impact Study Commission estimated that approximately 15 million people in the U.S. had gambling problems (National Opinion Research Center, 1999). By 2012, 31 states had conducted studies of gambling prevalence, with findings varying drastically, from a low of 0.6% in Delaware to a high of 8.1% in Puerto Rico (Williams et al., 2012).

Many personal, familial, and societal problems are associated with gambling addictions. Disordered gamblers often face problems in relationships, physical and mental health, employment, school, financial difficulties, and legal troubles (Dowling et al., 2016; Eby et al., 2016; Kalischuk et al., 2006; Latvala et al., 2019; Wan, 2012; Raisamo et al., 2013). Issues within the family include high divorce rates, child neglect, child abuse, family dysfunction, and a possible increase in intimate partner violence (Dowling et al., 2016; Kalischuk et al., 2006). Therefore, it is important to see how close and familial relationships impact the likelihood of developing Gambling Disorder.

### Problem Gambling and Gender

Gambling research has increasingly focused on the behavior of women and included gender as an explanatory variable. Some studies focus specifically on gender differences among gamblers that are seeking treatment (Echeburúa et al., 2011; Granero, et al., 2009; Malkin & Stacey, 2024; Potenza et al., 2002) or study only women’s gambling (Boughton & Falenchuk, 2007; Holdsworth et al., 2013; McCarthy et al., 2018; Nuske et al., 2016; Schull, 2002). There has also been a growth in research examining gender and gambling outside the U.S., such as Canada (Afifi et al., 2010; Faregh & Derevensky, 2013); Sweden (Abbott et al., 2018), France (Bonnaire et al., 2017), and Australia (Nuske et al., 2016). There have additionally been systematic reviews of research on gender and gambling behavior and/or gambling disorder (Gartner et al., 2022; Holdsworth et al., 2012; Merkouris et al., 2016). Despite the growth of research exploring various aspects of women’s gambling behaviors, few studies have included a theoretical understanding of gambling disorder focusing on gender.

The need to include gender in the study of gambling disorder is due, in part, to the expansion of legalized gambling throughout the U.S., which resulted in a larger percentage of women becoming frequent and problem gamblers (Gartner et al., 2022; LaPlante et al., 2005; McCarthy et al., 2018; Rogers et al., 2020; Volberg, 1994; Volberg, 2003). Rates of gambling disorder among women are increasing more rapidly than among men (McCarthy et al., 2018). Research concerning gambling and gender suggest that while women tend to start gambling later in life, they may advance to gambling disorder faster than men (Afifi et al., 2010; Crisp et al., 2000; Gartner et al., 2022; Hing et al., 2016; Nelson et al., 2006).

There are several important reported differences between women and men’s gambling that have been found based on gender. For example, female gamblers may gamble for different reasons, as women are more likely than men to gamble in order to “escape” problems in their life (Blanco et al., 2006; Gartner et al., 2022; Holdsworth et al., 2013; Sacco et al., 2011; Weatherly & Cookman, 2014; Schull, 2002), such as depression and similar mood disorders, loneliness, boredom, and stress (Blanco et al., 2006; Hing et al., 2016; Sacco et al., 2011).

While gender has been an increased focus of studies on gambling disorder, a review of the existing literature found that many holes still exist in understanding gender differences in gambling addiction (Gartner et al., 2022; Holdsworth et al., 2013; McCarthy et al., 2018). In addition, the differences that have been identified to date regarding men’s and women’s patterns in gambling behaviors suggest that men and women should be studied separately, not only to examine differences in gambling behavior but also to evaluate how different factors (such as strain) may provide different explanations based on gender. Based on the prior research detailed above, GST may be a good way to explain some of the suggested gender differences in gambling disorder.

### General Strain Theory (GST) and Problem Gambling

Strain theories were developed from the general belief that people may experience different types of strain, which can then lead to negative behavior, such as crime and addiction (Agnew, 1992). General Strain Theory (GST) was created as an adaption to Classical Strain by Robert Agnew (1992), offering new conceptualizations and negative emotions about the effects of strain. Among the changes in the theory, sources of strain could range in degree and type (Agnew, 1992; Agnew & White, 1992; Baron & Hartnagel, 1997; Ford & Schroeder, 2009). This allowed for greater possibility of the causes of strain and why people may resort to negative coping behaviors. GST also includes negative affective states as the result of strain, which may lead individuals into deviant behaviors. Emotions such as anger, disappointment, fear, and rejection can all occur due to strain.

Empirical support for GST has generally proven to be a valid theory for understanding addiction. For example, there has been some research showing that GST is effective in understanding other types of addictive behaviors, such as prescription stimulants, tobacco, alcohol, and marijuana, as well as eating disorders (Baron & Hartnagel, 1997; Ford & Schroeder 2009; Preston, 2004; Piquero et al., 2010). Based on these empirical assessments of GST, it seems suitable to a wide variety of addictive behaviors, including problem gambling.

While not specifically using GST in their research, some studies have found that the concepts embedded in GST are related to gambling behavior. For example, experiencing negative life events and/or stress may increase gambling behavior ( Aymamí et al., 2014; Bergevin et al., 2006; Elman et al., 2010; Felsher et al., 2010; Kaufman et al., 2002; Korman et al., 2008; Maniaci et al., 2016; McCormick et al., 2012; Santaella et al., 2013; Taber et al., 1987). Childhood maltreatment and abuse have been linked with gambling disorder (Felsher et al., 2010; Santaella et al., 2013). Similarly, trauma and daily stressors experienced by adults are strongly correlated with gambling problems (Elman et al., 2010; Kaufman et al., 2002; Taber et al., 1987). Likewise, gambling disorder severity has been linked to stress and negative life events occurring in childhood and adolescence (Bergevin et al., 2006; McCormick et al., 2012), and anger has been connected to the severity of gambling behavior (Aymamí et al., 2014; Korman et al., 2008; Maniaci et al., 2016).

Prior research has included GST to explain some aspects of problem gambling (Cheung, 2015; Eitle & Taylor, 2010; Greco & Curci, 2016; Man & Cheung, 2022; Tulloch et al., 2020). Only five published studies have specifically applied GST to gambling behavior. Using a sample of 2,248 participants from 262 families (where the primary recruitment was students attending a psychology course in Italy), Greco and Curci (2016) found a relationship between strain and both gambling and substance use. Eitle and Taylor (2010) determined that recent stressful life events could help predict gambling disorder in young adult male participants.

As stated above, Cheung’s (2015) study of 1,620 married couples in China found that strain experienced by a male partner could predict both his and his wife’s likelihood of developing into problem gambling, however, while strain by the female partner could predict her likelihood of developing a gambling problem, there was no association with her husband’s gambling behavior. In contrast, Man and Cheung (2022) utilized the same data to assess whether trying to adhere to traditional gender norms as a form of social strain could predict the likelihood of gambling disorder. Their multivariate analysis of gender and strain found that adhering to gender norms increased the effect of strain on gambling disorder among males who possessed more traditional gender characteristics than women. Essentially, they found that traditional gender norms increased men’s likelihood of developing gambling disorder when facing strain while serving as a protective buffer to prevent gambling disorder for women under strain. Although this study looked at gender, gambling, and GST, it focused on gender norms rather than the specific role of strain from another’s gambling based on gender.

A recent study by Kabiri et al. (2020) used a mediational model of GST to examine illegal sports gambling in Iran through a survey of 392 known gamblers (non-probability convenience sample). While the authors did not specifically examine gambling disorder (they studied participation in illegal gambling), they found a relationship between GST and gambling in that financial strain and control deficit could help explain participation in illegal sports gambling. While not specifically examining GST in relation to an individual’s gambling disorder, Tulloch et al. (2020) recently examined data from a general population sample of 15,475 Australian adults. They found that GST could help explain the relationship between negative behaviors (such as addictions and criminal behavior) of problem gambler’s family members.

This study will be one of the only studies that examines the relationship between strain and problem gambling using a general population data source in the United States, and one of the few to examine whether and how gender moderates the relationship between GST and problem gambling. Based on the likely relevant concepts from prior research and those contained in GST, it is hypothesized that strain from a perceived belief that a friend or family member gambles too much will be related to gambling disorder. Based on prior research, the expected direction is that strain from a friend or family member’s problem gambling will increase the prevalence of gambling disorder. Additionally, this study tests whether gender moderations the relationship between strain from the perceived belief that a friend or family member gambles too much and gambling disorder with the expected finding that strain will have a stronger relationship with problem gambling for women than men.

## Data & Methodology

The data analyzed in this study consist of the Baseline General Population Survey (BGPS) and the Baseline Online Panel Survey (BOPS) from the Social and Economic Impacts of Gambling in Massachusetts (SEIGMA) study. The SEIGMA study assessed the gambling behavior of the general public, motivations for gambling, prevalence of problem gambling (gambling disorder), social and health impacts of gambling, comorbidities with problem gambling, and general demographic backgrounds of the participants.

NORC performed data collection for the BGPS at the University of Chicago from September 2013 through May 2014. With a response rate of 36.6%, the BGPS used multi-mode (telephone (8%), online (40%), and paper/pencil (52%)) address-based sampling, where the adult with the most recent birthday at the address was selected as the survey respondent. Address-based sampling is a random sample from a listing of all residential addresses in Massachusetts.

Data collection for the BOPS was conducted by Ipsos Public Affairs from October 2013 to March 2014 utilizing a stratified sample (based on the U.S. Census) of Massachusetts by age, gender, and region. Ipsos continued to reach additional potential participants in the state until at least 5,000 surveys were completed. The SEIGMA study represents one of few recent available population-based data assessing problem gambling and gambling behaviors in the United States.

The BGPS and BOPS survey data were combined, resulting in a total of 14,624 participants. For this study, the sample includes only those individuals who gambled regularly in the past year, defined as gambling at least monthly. While problem gambling was not assessed for the majority of the original study participants, it was assessed for all individuals who stated that they gambled at least monthly, totaling 5,852 respondents. The focus on those participants who gambled at least monthly allowed for the examination of how strain may affect regular gamblers, focusing on how strain is related to gambling disorder.

Table 1 provides demographic information for each study sample. There were slight differences in demographics between the two samples. The mean age of BGPS participants was approximately 8 years older than BOPS participants, the BGPS had a slightly higher percentage of women (59% to 53%), and were more likely to be married, employed, and college-educated. BOPS participants were slightly more likely to be White. All demographics were made into dummy variables, allowing for a single regression analysis.

**Table 1:** Descriptive statistics on the samples, control, dependent, and independent variables

	BGPS	BOPS	Gambled at least monthly (Combined BGPS/BOPS)
	N = 9,578	N = 5,046	N = 5,852
Gambled monthly in past 12 months	35.0% (N = 3,355)	49.5% (N = 2,497)	
Mean age	55.2	47.0	52.6
Women	59.1%	52.9%	46.4%
Married	52.8%	44.2%	50.2%
FT Employed	57.3%	54.9%	57.6%
White (non-hisp.)	81.7%	85.2%	85.8%
College educ	53.6%	39.7%	39.1%
Problem gambler on PPGM	1.3% (N = 129)	6.3% (N = 317)	7.6% (N = 446)
Perceived problem gambling of a family member/friend level of strain			
0	(N/A)		81.1% (N = 4,747)
1	(low strain)		7.3% (N = 429)
2			2.1% (N = 123)
3			1.4% (N = 80)
4			1.5% (N = 90)
5			1.7% (N = 99)
6			1.1% (N = 64)
7			1.2% (N = 73)
8			0.8% (N = 46)
9			0.5% (N = 27)
10	(high strain)		1.3% (N = 74)
Relationship to perceived problem gambler			
Spouse/partner			1.9% (N = 113)
Parent/step-parent			2.1% (N = 123)
Child/step-child			0.8% (N = 46)
Other family			5.7% (N = 334)
Friend			6.0% (N = 350)
Other			2.9% (N = 167)
Not applicable			80.6% (N = 4,719)

### Dependent Variable

The SEIGMA study assessed problem gambling using two validated assessment tools: the Problem and Pathological Gambling Measure (PPGM) and the Problem Gambling Severity Index (PGSI). For this research, results from the PPGM are utilized. Williams and Volberg (2010; 2014) developed the PPGM to address weaknesses in other available measures. The PPGM assessed gamblers using 14 items broken into three sections (problems, impaired control and other issues) and scored gamblers into five potential categories: non-gambler, recreational gambler, at-risk gambler, problem gambler, or pathological gambler (Williams & Volberg, 2010). Utilizing results from the PPGM assessment, those who score as a problem gambler or pathological gambler were considered a “problem gambler” (or having gambling disorder) for the purpose of this study, as intended by the PPGM (Williams & Volberg, 2010; 2014). Based on the PPGM assessment, those who fall into these categories are most likely to face gambling-related harms, allowing for the best assessment of the potential relationship between strain and gambling disorder. Frequency and means for the PPGM are included in Table 1. Relevant to this study, 7.6% of the participants who gambled at least monthly scored on the PPGM as problem or pathological gamblers.

### Independent Variables

The primary variable from the SEIGMA survey used to assess the hypotheses was based on strain from another person’s perceived problem gambling. In addition to the question, the data includes the respondent answering, “Overall, on a scale from 1 to 10, how much has this person’s gambling affected you negatively during the last 12 months?” Strain from the perceived problem gambling of a family member or friend was measured using a Likert scale ranging from 1–10 (with a 1 indicating a low level of strain and 10 indicating a high level of strain caused by the other person’s gambling behavior). Those who did not have a family member or friend causing strain from their gambling were given a score of 0. This allows for assessment as an interval variable.

Descriptive statistics for the independent variables are included in Table 1. About 20% of the sample conveyed having a family member or friend who gambles too much, with the strain caused by the other person’s gambling generally not causing a high level of strain. Only about 3.8% of respondents indicated that the strain they experienced from someone else’s perceived problem gambling caused strain at a level of 7 or higher (out of 10).

Additionally, gender is used as an independent variable to assess whether gender moderates the relationship between problem gambling and strain. Approximately 46% of people reporting they gambled monthly were women, supporting prior research that women are engaging in frequent gambling at levels similar to men.

### Control Variables

To assess demographic differences in this study, several variables are used as control variables, specifically age, employment status, marital status, education, and race. Frequencies for all control variables are also included in Table 1. For the participants who gambled at least monthly, the participant mean age was in the early 50’s; about half of the participants were married, 39% college educated, 58% employed, and over 85% were White.

### Methods

Statistical analysis was conducted using SPSS software. Analysis began with bivariate analysis to identify preliminary relationships between the independent and dependent variables. Because the dependent variable in this study is binary, logistic regression was used to determine if the bivariate relationships hold after controlling for the remaining variables. Subgroup analysis was conducted to determine if the impact of strain on problem gambling differs for male and female respondents. Several logistic regression models were conducted to examine the relationship between the negative strain from someone else’s perceived problem gambling and problem gambling. I then conducted a subgroup analysis to determine whether gender moderates the relationship between strain and problem gambling.

## Analysis and Results

Independent-sample t-tests of all variables determined whether and how disordered gamblers and non-disordered gamblers differed and the difference between male and female respondents (Table 2). The bivariate analysis indicates that many variables differ significantly between those with gambling disorder and non-disordered gamblers. Starting with the demographic variables, disordered gamblers are younger than non-disordered gamblers by about 11 years and less likely to be college-educated or employed full-time. Those with gambling disorder are more likely to be non-white than non-disordered gamblers. There is no statistical difference between disordered gamblers and non-disordered gamblers regarding their marital status.

**Table 2:** Bivariate analysis of all variables by problem gambling status and gender

	All	Non-problem gambler (N = 5,406)	Problem gambler (N = 446)	*P*^a^	Men (N = 3,104)	Women (N = 2,715)	*P*^a^
Demographics							
Age	52.56	53.39	42.64	.000**	52.74	52.31	.334
White (non-hisp.)	0.85	0.86	0.74	.000**	0.85	0.85	.900
Coll. Ed	0.40	0.40	0.31	.000**	0.42	0.36	.000**
Married	0.59	0.58	0.60	.642	0.60	0.57	.013*
Employed	0.51	0.52	0.34	.000**	0.55	0.46	.000**
PPGM	0.08	–	–	–	0.10	0.05	.000**
Strain from another person’s gambling problem	0.69	0.55	2.32	.000**	0.63	0.76	.008*
Relationship type***							
N/A	–	0.94	0.06		0.53	0.46	
Spouse	–	0.68	0.32		0.38	0.62	
Parent	–	0.84	0.16		0.46	0.54	
Child	–	0.85	0.15		0.44	0.57	
Other family	–	0.87	0.13		0.47	0.53	
Friend	–	0.88	0.12		0.61	0.39	
Other	–	0.78	0.22		0.59	0.41	

*p < 0.05 **p < 0.01

*** Due to nature of variable, a Chi-Square was conducted to determine relationship type

There is a large difference between non-disordered gamblers and those with gambling disorder in terms of the strain they feel based on how they perceived someone else’s problem gambling. While non-disordered gamblers scored a mean of 0.55 on the 0–10 scale, disordered gamblers reported significantly greater strain with a mean score of 2.32. A chi-square test for independence indicated a significant association between problem gambling and the type of relationship with the other person based on gender, χ^!^ (6, n = 5,819) = 0.07, *p* = 0.000, *phi* = 0.07), and with a gambling problem, χ^!^ (5, n = 5,852) = 0.189, *p* = 0.000, *phi* = 0.189) (Table 2). Based on the analysis, having a spouse/partner that one perceives to gamble too much appears to be the strongest relationship associated with the respondent’s own problem gambling. As for gender, women have higher values in all relationships, especially spouse (62% of respondents who have strain from their spouse’s problem gambling were women), while men have higher values toward a friend (61%) or some other relationship with the problem gambler (59%).

Moving to the analysis broken down by gender, there are several statistically significant differences between men and women. Men in this sample of regular gamblers are more likely to be college-educated, employed full-time and married than women. There are no statistically significant differences in the respondents based on gender in terms of age or race. Men are twice as likely than women respondents to score as problem gambler on the Problem and Pathological Gambling Measure (PPGM) (10% of men are classified as disordered gamblers compared to 5% of women), consistent with prior research. This analysis also indicated a strong relationship between experiencing strain from another person’s perceived gambling problem (reported on a 0–10 scale) and gender, with women reporting a higher level than men.

### Strain from Another Person’s Perceived Gambling Behavior

Cheung’s (2015) study found a relationship between the strain caused by a spouse’s problem gambling on the likelihood of the spouse being a problem gambler. In order to assess whether similar strain has a relationship with problem gambling in the U.S., analysis is conducted to assess whether strain from another person’s perceived problem with gambling (assessed on a 0 (no strain) through 10 (high strain) Likert scale) is related to problem gambling and whether strain from specific relationships (especially that of spouses) has a greater likelihood for a relationship with problem gambling than others.

Logistic regression was performed to determine whether stress from another person’s gambling impacted problem gambling (Table 3). The full model containing all variables is statistically significant and explains 22.3% of the variation in problem gambling. As shown in Model 1, three independent variables make a unique statistically significant contribution to the model (age, gender and strain from another person’s perceived problem gambling). The strongest predictor of problem gambling is gender at an odds ratio of 2.43 (1/0.410). Strain from another person’s perceived problem gambling is also significant, with an odds ratio of 1.307. This seems to suggest a relationship between strain from someone else’s perceived problem gambling and the individual 4being a problem gambler, supporting the study hypothesis.

**Table 3:** Logistic regression predicting likelihood of problem gambling with strain from another person’s gambling

	Model 1: Full model (N = 5,852)			Model 2: Based on gender					
				Women (N = 2,715)			Men (N = 3,104)		
	*B*	*SE*	*Exp(b)*	*B*	*SE*	*Exp(b)*	*B*	*SE*	*Exp(b)*
Constant	− 975*	.370	.377	− 2.101**	.577	.122	− 835	.486	.434
Women	− 892**	.192	.410	–	–	–	–	–	–
Age	− 021*	.006	.979	− 019	.010	.981	− 023*	.008	.978
White	− 321	.214	.725	− 009	.357	.991	− 516	.273	.597
Coll ed	− 292	.209	.746	− 397	.353	.672	− 264	.263	.768
Married	− 322	.202	.725	− 230	.321	.795	− 365	.263	.694
Employed	− 101	.199	.904	− 310	.309	.733	.025	.262	1.025
Strain from another person’s gambling	.268**	.030	1.307	.273**	.047	1.314	.269**	.039	1.308
Pseudo R-square (Nagelkerke)	.223			.183			.232		

*p < .05; ** *p* < .001

The dataset was then split based on gender to see whether gender acted as a modifying variable between strain from another person’s perceived problem gambling (Model 2). For men, two variables are significant (age and strain from another person’s gambling) and for women only strain from another person’s gambling is significant. For both male and female problem gamblers, the strain from another person’s gambling had significant odds ratios (men: odds ratio = 1.308; women odds ratio = 1.314). Paternoster’s Z scores were calculated to see whether the coefficient difference was significant based on gender. The difference in the coefficients is not significant, for women *B* = 0.273, *SE* = 0.047; for men *B* = 0.269, *SE* = 0.039; *z* = 0.065, indicating that there is not a difference based on gender in the relationship between problem gambling and strain from someone else’s perceived gambling.

In order to test whether these data support Cheung’s (2015) findings regarding strain from spouse’s gambling, a one-way between-groups analysis of variance (ANOVA) is estimated to explore the impact of type of relationship on problem gambling (Table 4). There is a statistically significant difference at the *p* < 0.05 level for different types of relationships: F (5, 5852) = 1560.17, *p* = 0.000. Post-hoc comparisons using the Tukey HSD test indicated that the mean score between all groups were significantly different. The relationship with a spouse or partner who is a problem gambler shows the largest negative strain (mean = 5.61).

**Table 4:** ANOVA comparing types of relationships with problem gambler’s and level of negative strain

	All (N = 1,133)			Women (N = 542)			Men (N = 586)		
	N	Mean	S.D	N	Mean	S.D	N	Mean	S.D
Spouse	113	5.61	3.08	69	5.25	3.08	43	6.21	3.05
Parent	123	4.41	2.98	66	4.14	2.96	57	4.74	3.01
Other fam	380	3.37	2.89	200	3.71	2.98	177	2.99	2.75
Friend	350	2.84	2.51	137	2.96	2.60	211	2.76	2.46
Other	167	3.29	3.29	69	3.86	3.33	98	2.88	2.72

The file was then split based on gender and a Two-Way ANOVA was conducted to compare relationship types with perceived problem gambling (Table 4). There continues to be a statistically significant difference at the *p* < 0.05 level for the different types of relationships for both men and women: [men: F(5,3104) = 835.24, *p* = 0.000; women: F(5, 2715) = 744.13, *p* = 0.000]. Spouse continues to be the relationship with the largest impact. This relationship has a larger impact on men (mean = 6.21) than women (mean = 5.25).

Logistic regression was then performed using five of the six demographic variables (marital status is not included because it correlated with strain from a spouse’s problem gambling). Relationship type was made into a dummy variable based on whether the relationship causing strain was by a spouse/partner (Table 5). For all participants, the regression is statistically significant and explained about 12% of the variation in problem gambling.

**Table 5:** Logistic regression predicting likelihood of problem gambling with strain from spouse/partner gambling

	Model 3: Full model (N = 5,852)			Model 4: Based on gender					
				Women (N = 2,715)			Men (N = 3,104)		
	*B*	*SE*	*Exp(b)*	*B*	*SE*	*Exp(b)*	*B*	*SE*	*Exp(b)*
Constant	.181	.188	1.199	− 589	.294	.555	.150	.233	1.162
Women	− 759*	.111	.468	–	–	–	–	–	–
Age	− 039**	.003	.969	− 035*	.006	.966	− 041**	.004	.960
White	− 378	.125	.685	− 417*	.212	.659	− 343*	.157	.710
Coll ed	− 377*	.116	.686	− 291	.204	.748	− 427*	.142	.563
Employed	− 216	.112	.806	− 424*	.188	.654	− 087	.141	.917
Strain from spouse gambling	1.901**	.224	6.693	1.460**	.335	4.304	2.427**	.332	11.321
Pseudo R-square (Nagelkerke)	.124			.084			.133		

^*^p < .05; ** *p* < .001

Age, college education, gender, and strain from spouse/partner gambling are all significantly related to problem gambling. Strain from spouse/partner’s problem gambling shows the strongest relationship with an odds ratio of 6.693, supporting prior research that strain from spousal problem gambling is related to a person’s gambling problem. Because large odds ratios may be a sign of multicollinearity, VIF values were calculated and all were less than 1.5, indicating no multicollinearity.

In order to compare the relationship based on gender, the data were split and recalculated for men and women (Model 4). Including strain from a spouse’s gambling explains 8.4% of the variation in problem gambling for women and 13.3% for men. Both men and women have four significant variables, with strain from a spouse’s problem gambling having the largest odds ratio. For women the odds ratio is 4.304 and for men the odds ratio is 11.321. Due to the high odds ratios, multicollinearity was checked and all VIF values were under 1.5 for both men and women, indicating no multicollinearity.

Paternoster’s Z scores were calculated to see whether the coefficient difference was significant based on gender. The difference in the coefficients is significant, for women *B* = 1.460, *SE* = 0.335; for men *B* = 2.427, *SE* = 0.332; *z* = -2.05 indicating that there is a difference based on gender in the relationship between problem gambling and strain from a spouse’s gambling. In contrast to Cheung’s (2015) findings, in this study men’s problem gambling is better explained by strain from a spouse’s problem gambling than for women. Additionally, unlike Cheung’s (2015) findings, strain from a spouse’s problem gambling is significant for both men and women.

## Discussion & Conclusions

This study explored two research questions to advance knowledge of the relationship between GST and problem gambling. Drawing on prior research, the included forms of strain were expected to have a relationship with problem gambling (Cheung, 2015; Eitle & Taylor, 2010; Greco & Curci, 2016; Man & Cheung, 2022). This was especially true when examining strain from perceived spousal problem gambling based on results from prior studies (Cheung, 2015; Greco & Curci, 2016). Findings from this research support prior research that strain caused by a spouse/partners perceived problem gambling may have a relationship with problem gambling.

The second research question examined whether gender moderated the relationship between strain and problem gambling, and if so, in what ways. Based on prior research concerning why women gamble and may become problem gamblers, women were expected to have a stronger relationship between strain and problem gambling than men (Blanco et al., 2006; Hing et al., 2016; Sacco et al., 2011). In this study, however, when gender differences were found, men generally had stronger associations between strain and problem gambling than women.

This study found a strong relationship between the level of strain the respondent had from another person’s problem gambling and the respondent being a problem gambler. This was true for men and for women and applied to many types of relationships (friend, family member, and spouse). For both men and women, the strongest relationship to have this link was when their spouse/partner was perceived to have a gambling problem. In this study, men have a stronger relationship between their problem gambling when they have strain based on the perception that their spouse/partner has a gambling problem than women. These findings seem to support Cheung’s (2015) study of married couples in China that strain from the spouse’s gambling could predict both men’s and women’s problem gambling, however men having the stronger effect is a new finding from this study. Unlike Cheung’s (2015) study, however, the current study did not pair up married couples; there is no ability to test whether strain by one partner could predict their specific spouse’s gambling behavior.

*Gender* Unsurprisingly, gender had with problem gambling, however, gender played only a minor role in understanding the relationship between GST and problem gambling in this study. Prior research on GST and problem gambling has not extensively considered gender. It was anticipated in the current study that women disordered gamblers would have greater levels of strain than men, however, this was not the finding indicating that other forms of strain or other factors likely have a stronger relationship to their problem gambling than strain caused by another’s gambling. Men, not women, had a slightly greater relationship between strain from their spouse/partner’s perceived problem gambling and their own problem gambling. These findings suggest that gender may not generally be a moderating factor between GST and problem gambling, except possibly for men based on their perception of their spouse’s problem gambling.

*Demographic Variables* Demographic variables are discussed here briefly, as most studies examining problem gambling have not been based on large population-based data and findings in this research may help inform whether prior demographic findings based on problem gambling, as well as gender differences, remain constant when using population-based data. This study found mixed results among the relationship between several demographic criteria and problem gambling, especially based on gender. According to this study, disordered gamblers averaged over a decade younger, were more racially diverse, had less college education, and were less likely to be married than non-disordered gamblers. Employment status was not related to problem gambling status.

When comparing the demographics by gender among disordered gamblers, only age remained a significant demographic for women, where women with gambling disorder continued to be about a decade younger than non-disordered gamblers. This differs from prior research indicating that women tended to be older (Afifi et al., 2010). Men were about twice as likely to be disordered gamblers than women, supporting prior research that men are more likely to be problem gamblers. There were many more differences for men, where younger age, non-white race/ethnicity, marital status (unmarried), and employment (less full-time employed) were all significantly related to disordered gambling. Such findings support the need to continue to examine problem gambling demographics based on gender.

### Implications

For the past 30 years, many studies have referred to women as primarily “escape” gamblers, trying to hide from problems (potential sources of strain) in their lives, and men as “action” seeking gamblers (Blanco et al., 2006; Sacco et al., 2011; Weatherly & Cookman, 2014). The current study conversely suggests that men and women may both have a relationship between at least some types of strain and disordered gambling and may, therefore use gambling as an “escape.” More studies that compare findings based on gender are needed, including an examination of whether descriptions of women as primarily “escape” gamblers and men as primarily “action” gamblers continue to be true. Studies should examine whether men and women continue to have a relationship between certain kinds of strain and whether they choose to gamble to “escape” feeling these strains.

In terms of gender-specific inclusion, this study was limited by the binary gender data in the study. This limited the account for individuals not identifying specifically on the binary gender spectrum. For example, in the analysis of strain caused by spousal/partner perceived problem gambling, the spouse/partner’s gender identity is unknown. With so few studies concerning problem gambling including LGBTQ + data, it is important that future studies include the ability to account for diverse identities.

Additionally, a study in the U.S. that delves deeper into the relationship between strain from a spouse’s problem gambling and the other spouse also being a problem gambler is also warranted. The current study could not examine whether strain by one partner could predict the likelihood of developing gambling problems based on gender, as Cheung’s (2015) study found. Since this study did find that strain from a spouse’s gambling was related to disordered gambling, especially for men, a future study is warranted to see if Cheung’s (2015) overall findings are consistent for couples in the U.S.

This study focused on strain from the perception of another person’s problem gambling to see whether there was a relationship between GST and problem gambling. Results show a relationship and where differences were found based on gender, men in this study had stronger relationships between strain and disordered gambling. These are important findings and encourage future researchers on gambling-related harms and gambling disorder to compare results based on gender.

## Data Availability

The data that support the findings of this study are available from Massachusetts Gaming Commission but restrictions apply to the availability of these data, which were used by permission for the current study and so are not publicly available. The data are, however, available from the authors upon reasonable request and with the permission of the Massachusetts Gaming Commission.

## Footnotes

1 There are many terms used interchangeably for gambling addiction, including problem gambling, severe problem gambling, pathological gambling, compulsive gambling, and/or gambling disorder. For the purpose of this research, I will use *problem gambling* and Gambling Disorder interchangeably as the terms for harms related to gambling addiction.
